# Factors Associated with Burnout Syndrome in Primary and Secondary School Teachers in the Republic of Srpska (Bosnia and Herzegovina)

**DOI:** 10.3390/ijerph17103595

**Published:** 2020-05-20

**Authors:** Nada Marić, Stefan Mandić-Rajčević, Nataša Maksimović, Petar Bulat

**Affiliations:** 1 Faculty of Medicine, University of Belgrade, 11000 Belgrade, Serbia; drnadamaric@gmail.com (N.M.); petar.bulat@med.bg.ac.rs (P.B.); 2 Institute of Occupational and Sports Medicine of the Republic of Srpska-Center Bijeljina, 763000 Bijeljina, Republic of Srpska, Bosnia and Herzegovina; 3 School of Public Health and Health Management and Institute of Social Medicine, Faculty of Medicine, University of Belgrade, 11000 Belgrade, Serbia; 4 Institute of Epidemiology, Faculty of Medicine, University of Belgrade, 11000 Belgrade, Serbia; natasa.maksimovic@med.bg.ac.rs; 5 Serbian Institute of Occupational Health, 11000 Belgrade, Serbia

**Keywords:** occupational stress, education personnel, behavior type, work-life balance

## Abstract

*Objectives*: The aim of this study was to estimate the prevalence of burnout syndrome in a large sample of primary and secondary school teachers in the Republic of Srpska (Bosnia and Herzegovina) and identify the factors associated with burnout in this population. *Methods*: This cross-sectional study was conducted in August and September of 2018, on a sample of 952 teachers. Beside socio-demographic information, Bortner scale, Job Content Questionnaire, and Maslach Burnout Inventory were filled in by the study participants. *Results*: Only 5.1% of teachers reported high levels of emotional exhaustion, 3.8% reported high levels of depersonalization, and 22.3% reported low levels of personal accomplishment. Behavior type, specifically type-A behavior, was associated with higher levels of emotional exhaustion. The most important factors associated with burnout were work–life characteristics and job-demand-control model of occupational stress. *Conclusions*: Our study shows a low prevalence of emotional exhaustion and depersonalization in teachers in the Republic of Srpska before the beginning of the new school year. Since similar studies show a high prevalence of burnout at the end of the school year, a potential seasonality of this syndrome should be considered and explored further.

## 1. Introduction

Burnout syndrome has been in the focus of research since the 1970s as a stressogenic interpersonal reaction at the workplace, defined by three dimensions: emotional exhaustion (EE), cynicism/depersonalization (DP), and reduced personal accomplishment (PA) [1]. The research of this syndrome came into focus primarily due to the consequences it can produce on the health of the workers, leading to an economic cost for the employers and the country itself [1,2,3,4]. In Norway, it was estimated that the annual costs of this phenomenon could reach 1.7 billion euro [5]. In Germany, stress-related disorders are considered the leading cause of early retirement [6].

Working environment factors are a significant contributor to burnout syndrome. Several European countries even recognize burnout syndrome as an occupational disease [5]. On the other hand, not all employees sharing the working environment develop burnout syndrome, which underlines the influence of personal characteristics [7]. Among the socio-demographic characteristics of the employees, gender, gender equality, marital status, education, and experience are believed to have the highest impact, although conflicting data are published regularly [2,8,9,10,11,12,13]. Work-life conflict, as well as social support, received and perceived, may have a significant role in the development of burnout syndrome [14,15].

Early research on burnout indicated that this syndrome is predominant in the services sector—such as healthcare, social services, and mental health, as well as education—due to the intensive work with people and high emotional challenges. Later, authors noticed a high prevalence of burnout in occupations that include high job demand and time pressure (e.g., managers) [1,4]. A systematic review of prospective studies provided evidence that burnout syndrome is associated with many physical and mental disorders resulting in different occupational consequences (job dissatisfaction, absenteeism, presenteeism, disability pension) [3]. Among various occupations commonly affected by burnout, few are considered of such socio-economic importance as school teachers. In this population, burnout syndrome requires more attention due to its influence on the educational process, mental, and emotional development of children, as well as potential long-term consequences such as students’ academic outcomes [16,17]. A review and meta-analysis has shown that countries differ in the prevalence of burnout and even burnout scales [2]. Despite these differences, having in mind that teaching is an occupation that provides an essential service, burnout in this population is a health issue that must be addressed across countries.

Bosnia and Herzegovina (BiH) was affected by a brutal civil war from 1991 to 1995 after the break-up of Yugoslavia and later divided by ethnic groups in two entities and one district. Social systems and infrastructure, including education, were damaged or destroyed [18]. BiH consists of two entities: Federation of BiH and Republic of Srpska. In the Federation of BiH, predominant populations are Bosnian (Muslim) and Croatian (Catholic), while in the Republic of Srpska the population is mostly Serbian (Orthodox). Until now, only a small study in school teachers has been performed in the Republic of Srpska indicating a low percentage of burnout syndrome in this population and contradicting previously published studies as well as indicators of increased stress in this population due to the conflicts, political, national, and religious tensions [19].

The aim of this study was to estimate the prevalence of burnout syndrome in primary and secondary school teachers in the Republic of Srpska, identify the factors associated with burnout syndrome, and recommend preventive actions.

## 2. Materials and Methods

This cross-sectional study was done in August and September of 2018 in all of the six administrative regions of Republic of Srpska (Prijedor, Banja Luka, Doboj, Bijeljina, East Sarajevo, and Trebinje). The questionnaires were filled during regular health check-ups of school teachers, which were done in the Institute for Occupational and Sports Medicine in Banja Luka, regional Occupational Health Centers (Prijedor, Doboj, Bijeljina, Trebinje), and field visits done in East Sarajevo. Participation in the study was on a voluntary basis.

### 2.1. Study Population and Sample

The school education in BiH starts with the obligatory primary (or elementary) school from the age of 6 and up to the age of 13 and continues to secondary (high school) education from the age of 14 and up to the age of 18. The official data for the school year 2017/2018 shows there are 11,893 teachers in the Republic of Srpska (68.37% female). The official number of primary school teachers is 8122 (70.89% female), while the number of secondary school teachers is 3771 (44.61% female). Medical examinations are mandatory prior to the beginning of the school year. Teachers who were on sick leave were also included in the study. Participation in the study was proposed to a randomly selected sample of teachers, namely while accessing the administrative part of the examination, one out of every four teachers was invited to fill in the questionnaire. A total of 1176 questionnaires were distributed, and 1002 (85.2%) were collected at the end of the study period. Another 50 questionnaires were excluded from the study due to a large percentage of missing data. The final response rate was 80.95%, and our sample represents around 8% of the teachers’ population in the Republic of Srpska.

### 2.2. Data Collection

The socio-demographic data of teachers was collected using a general questionnaire. This questionnaire was designed specifically for this study based on a preliminary interview with a sample of school teachers. Beside standard socio-demographic characteristics (gender, age, marital status, number of children) it included variables regarding the characteristics of the working environment: years of service as a teacher, employment in primary and/or secondary school, type of contract (fixed-term or indeterminate), overtime work, as well as variables regarding teacher’s satisfaction with equipment at the workplace and salary (five-step Likert scale). In addition, it included variables regarding their living environment, such as satisfaction with social support from family and/or friends and the existence of work–life conflict. We assessed the presence of work-life conflict using a single item: “In the last three weeks, how often were you annoyed or upset because of the inability to reconcile work with family and/or partner commitments?“ proposed by Roberts (2014) [20].

#### 2.2.1. Bortner Scale (BS)

Behavior type was assessed using the linguistically adapted Serbian version of the Bortner scale (BS) (1969) [21]. This 14 item scale has been widely used [22], with a score of 84 or above classified as Type A behavior, and a score of 14–83 as Type B behavior. Previous research has demonstrated that this measure has adequate reliability and construct validity [23].

#### 2.2.2. Job Content Questionnaire (JCQ-Karasek) 

Job Content Questionnaire (JCQ) is commonly used in the assessment of job-related stress [24,25,26,27]. It was translated and validated by the Serbian Institute of Occupational Health’s Dr. Dragomir Karajović, and it measures two constructs: psychological demands and job control. Job control has two subscales: skill discretion and decision authority. Additional scales such as social support, which is believed to act as a mediator of work stress, have also been developed. All JCQ scores were calculated using official formulae available in the paper describing the overall methodology [28]. Job strain was calculated as the ratio of demands to control [24].

#### 2.2.3. Maslach Burnout Inventory-Human Service Survey (MBI-HSS)

Burnout syndrome was assessed using the widely used and accepted Maslach Burnout Inventory-Human Services Survey (MBI-HSS) which has 22 items and measures the three dimensions of the burnout syndrome: emotional exhaustion (EE), depersonalization (DP), and a low sense of personal accomplishment (PA) [1]. We used the Serbian version of the MBI-HSS [29].

### 2.3. Statistical Analyses

Continuous variables were first plotted, and if necessary, the Shapiro-Wilk test was performed to verify the normal distribution. In case of normally distributed variables, mean and standard deviation are shown in the tables, while the differences between groups are tested using the *t*-test, in case of two groups, and ANOVA in case of more than two groups. For variables not falling under the normal distribution, median, minimum, and maximum values are reported in tables, while the differences between groups are tested using the Mann-Whitney-Wilcoxon test for two groups, or Kruskal-Wallis test in case more than two groups are present. Categorical variables are presented by the number of observations and the percentage within the group in parenthesis (absolute and relative frequencies). The chi-squared test is used to compare frequencies between groups, or Fisher test when the expected frequency is less than 5 in one of the cells. In the case of more than two groups, pairwise comparison adjusting for multiple testing is also performed (Tukey in case the row-variable is normally distributed or Benjamini-Hochberg method if this condition was not met). The MBI-HSS score was treated as the dependent variable in the multiple linear regression model. We assessed the association between burnout and independent variables, namely the gender, age, marital status, number of children, length of service, workplace, work contract, overtime work, existence work-life conflict, self-assessment with material satisfaction, satisfaction with equipment at work and support by family and friends, Karasek’s stress model at work, and behavior type. Statistical data analysis was done using the IBM SPSS Statistics 25 software (IBM, Armonk, NY, USA).

### 2.4. Ethical Approval

All participants in our study received a leaflet with detailed information regarding the study goals and were informed that the study is completely anonymous and that they do not have to provide any personal information. Participation in the study was on a voluntary basis, and due to the concerns of the workers regarding their honest answers about their employer, the working conditions, and their own mental wellbeing, the authors proposed in the study protocol that no written informed consent is asked. The Ethical Committee of the Institute for Occupational and Sports Medicine Republic of Srpska approved the study and the omission of the written informed consent (No. 01-24/18, 20.11.2018).

## 3. Results

This study included 952 teachers, of which 250 (26.3%) were male and 701 (73.6%) were female (one study participant did not answer the question about gender). A small proportion of teachers (7 teachers) worked in both an elementary as well as a high school, and they were excluded from analyses that consider the workplace. All individual and socio-demographic characteristics of teachers participating in this study, stratified by gender and workplace, are presented in Table 1.

Occupational and work/life characteristics stratified by gender, workplace, and behavior type are presented in Table 2.

### 3.1. Job Content

A total of 13.9% of teachers indicated having job strain. Results of the job content questionnaire (Karasek) stratified by gender, workplace, and behavior type are presented in Table 3.

### 3.2. Burnout Syndrome

A small percentage of teachers reported high levels of emotional exhaustion (5.1%) and depersonalization (3.8%). Low levels of personal accomplishment were reported much more commonly (22.3%). A larger percentage of type-A teachers than type-B teachers (59% compared to 40%, respectively) reported emotional exhaustion, and this difference was statistically significant (*p* < 0.05). The frequencies of burnout in the study population, stratified by personal and socio-demographic characteristics are reported in Table 4.

Table 5 shows the scores of burnout syndrome stratified by work/life and occupational characteristics of the population.

Due to a large number of variables potentially explaining the amount of variance in the burnout scales, hierarchical linear regression analysis was used with four blocks representing the behavior type, socio-demographic characteristics, work–life characteristics, and occupational stress variables. First, the unmodifiable blocks were entered (behavior and socio-demographic characteristics), followed by modifiable factors (work–life characteristics and occupational stress). Detailed results of the hierarchical linear regression analysis are summarized in Table 6.

## 4. Discussion

This is a first large study that included almost 10% of all teachers in the Republic of Srpska (Bosnia and Herzegovina) measuring the prevalence of burnout syndrome and identifying factors associated with its dimensions.

The study population included a larger proportion of female teachers than male teachers, which is similar to other studies [10,13,27]. There was a statistically significant difference in the gender distribution between primary and secondary school teachers, with more male teachers in secondary schools. This can be explained by the number of technical (craft) secondary schools where male teachers traditionally teach many different subjects. Similar results were found in Swedish schools [10]. Most of the teachers included in the examined group of BiH (Republic of Srpska) teachers were younger than 45, similar to Chinese teachers [27], but different from Germans teachers [13]. A larger percentage of women teachers were divorced or widowed, which can be explained by the impact of war during the 90 s.

According to Karasek’s “Demand-Control model”, the strain is the result of a common effect of high psychosocial demands of the workplace and a low possibility of controlling or autonomy of workers at the workplace [28]. In a study in Lithuania [24], almost half of the teachers (47.4%) reported job strain; and in the examined group of BiH teachers (Republic of Srpska), it was only 13.9%. The prevalence of stress among teachers documented earlier in England was around 19.9−30.7%, and in secondary school teachers up to 36.6% [30]. Based on these results, we could assume that teachers in other countries may be exposed to greater psychosocial efforts, or that they are given less opportunity to work independently in the workplace. The results of studies conducted in China are in favor of our assumption. They reported (mean and SD) in the job demand scale of 33.92 (4.46), and in the job control scale 63.94 (8.31), while our results were 28.36 (4.71) and 69.73 (7.59), respectively [27].

In the Republic of Srpska, 5.1% of teachers reported high levels of emotional exhaustion (EE), 3.8% reported high levels of depersonalization (DP), and 22.3% reported low levels of personal accomplishment (PA). The study conducted in Lithuania found high EE in 25.6%, high DP in 10.6%, and low PA in 33.7% of teachers [24]. In Sweden, high EE was found in 36% of teachers, high DP in 11%, and low PA in 21% of cases [10]. In other European countries, the prevalence of burnout syndrome was also higher than in our sample, estimated to be between 25−30% [31].

An important fact to consider might be the time of the year of the study. Among published studies reporting the time of the (school) year when they were conducted, a majority were carried out in the middle of the school year or at the end of the school year [24,32,33]. On the other hand, the data from our study were collected just before the start of the new school year, after the summer break. One study carried out in November/December shows only slightly higher scores than ours (emotional exhaustion 11.98 (7.40), cynicism 5.50 (4.37), and professional efficacy 26.85 (8.35) [27]), but lower than those from studies carried out at the end of the school year. These differences between the beginning, middle and the end of the school year, suggest a possibility of seasonality as a characteristic of burnout in school teachers during their yearly work cycle, indicating that teachers might have higher burnout scores at the end of the school year. On the other hand, even after the summer break, 5% of our teachers started the school year with emotional exhaustion, 3.8% with depersonalization, and 22.3% with the feeling of low personal accomplishment. After inevitably being exposed to stress and other factors associated with burnout syndrome, they might be at higher risk of developing severe consequences of burnout.

Our results show that people with type-A behavior have higher levels of emotional exhaustion, contrary to a meta-analysis which indicated that type A behavior is related only to personal achievement [7]. Type-A behavior is characterized by high speed, ambition, and competitiveness, which is why these persons have a negative perception of the environment and negative response from the society, and our results have shown that they more often perceive stress, in line with other studies [23,34]. Other authors have suggested that type-A behavior teachers tended to report heavier workloads without actually having heavier workloads [35].

Various studies have indicated that women are more inclined to respond to emotional stress due to the traditional role of women in society [36], but in the present study, gender was not associated with burnout syndrome. In addition, our results have shown no difference in the work-life conflict between women and men, and work-life conflict was significantly associated with all three dimensions of burnout syndrome in all participants. Having in mind that women in our population were more satisfied with the support of the family or friends, which is a resource women tend to utilize more according to the literature, they may not have been more vulnerable to burnout than men and managed to answer both demands of work and family [37]. It is important to note that the work-life conflict question was addressed at the three previous weeks, which, due to the moment at which our study was done (before the beginning of the school year) included no or little work with students and no teaching activities. Therefore, the measured work-life conflict in our study cannot be considered representative of that experienced in the middle of the school year.

Among teachers of the Republic of Srpska, older teachers and those with more extended work experience had higher EE scores, which is in line with previous studies [13,38]. For example, Kamtisos (2018) suggests that after a certain number of years, the demands of work are more difficult to tolerate. However, in a survey conducted in China [27] and Greece [8], the youngest teachers were the most emotionally exhausted. Authors explain these results by older teachers developing adequate and effective defense mechanisms for coping with constant stress over time. Our results show that teachers in secondary schools in the Republic of Srpska have higher EE scores than teachers in primary schools. This can be explained by the fact that it is more demanding to work and establish a work atmosphere with teenagers than with small children. Similar results were found in a Swedish study [10].

Contrary to socio-demographic characteristics that cannot be influenced, the importance of work-life balance and professional characteristics opens the door for interventions that could prevent burnout in teachers. The seasonality of burnout levels, i.e., those described in our study at the beginning of the school year compared to those found in the literature, could become a basis for the development of regular screening programs for burnout syndrome, which could be implemented before the beginning, in the middle and at the end of the school year. Those identified at risk at the beginning of the school year, unable to recover during the summer break, are candidates for secondary and tertiary prevention measures, while those not at risk at this point could benefit from primary prevention measures throughout the school year. According to our multivariate model (see Table 6), psychosocial demands of work and skill discretion were significantly associated with all three dimensions of burnout. Supervisor support was also associated with burnout dimensions, possibly playing a role in the work-stress relationship, although low standardized β coefficients and the cross-sectional design of this study warrant caution in the interpretation of these results. Nevertheless, a previous study suggests that supervisor support is a moderator of burnout, and where present, it increases the resources of a teacher, making them able to face more psychosocial demands without stress [39]. Some authors even indicated that behavior patterns could be changed and developed guidelines for this aim. In cardiology, participants who followed behavior guidelines combined with a program concerning standard risk factors had a significant reduction in their type-A scores and subsequent lower recurrence of coronary heart disease [40], and this approach might be applicable to burnout.

There are several weaknesses in our study. The cross-sectional design of the study limits the interpretation of the causality of various factors identified in the pathogenesis of burnout syndrome. Bortner’s scale, used in this study to determine the behavior type, does not measure all of the individual characteristics—such as personality, temperament, affect, self-esteem, or locus control—which have shown a strong connection with burnout syndrome. Nevertheless, it is easily applied and requires no supervision by a trained psychologist, which makes it valuable in large occupational studies. The different aspects of culture on the perception of stress and burnout dimensions have not been examined to the detail, but the complexity of different national education systems requires perhaps a multicenter study and a different approach. Our study did not take into account the influence of students’ and parents’ behavior, possible violence against teachers, nor the size of the class, which was identified as a significant factor contributing to the development of burnout [8,24]. The “effort-reward” model, which suggests that teachers might tolerate even greater psychological demand if they were adequately rewarded, was not fully covered by our study [41]. Finally, the work-life balance question was addressed at the period without regular stressors and is not representative of the conditions during the school year, although it is interesting that it was found significantly associated with burnout even at this point in the schoolteachers’ work cycle.

Nevertheless, we managed to gather a large sample of teachers which matches, by gender, age, and geographical distribution, the teachers of the Republic of Srpska. The instruments used in our study are validated and reliable and have already been translated and used in the region. The number of factors potentially associated with burnout syndrome examined by our study, although not exhaustive, was large enough to offer valuable input for future studies and preventive measures.

## 5. Conclusions

Teachers in the Republic of Srpska (Bosnia and Herzegovina) rarely report high levels of emotional exhaustion and depersonalization, but often report low personal accomplishment. Type-A behavior is associated with emotional exhaustion, and teachers reporting higher job strain and work-life conflict (even measured at the moment without regular teaching activities) have higher scores on the emotional exhaustion and depersonalization scales, and lower scores on the personal accomplishment scale. Burnout was significantly associated with work-life characteristics and the job demand-control model of occupational stress, while socio-demographic variables and behavior types were less significant. Our findings indicate an interplay between the behavior type, job strain, and work-life conflict, and job demand-control model in developing burnout in teachers. The low levels of burnout found before the beginning of the school year in our study point toward the potential seasonality of the burnout syndrome, which should be further explored, and the identified variables associated with burnout can help develop preventive measures for burnout among school teachers. There is a need for a systematic nationwide study identifying and exploring a wide range of individual factors contributing to burnout, while also measuring environmental and intrinsic job features.

## Figures and Tables

**Table 1:** Individual and socio-demographic characteristics of teachers stratified by gender and workplace.

Study Sample Characteristic’s *N* (%)		All	Gender *N* = 951			Workplace (School Type) *N* = 945		
		*N* = 952	Females *N* = 701	Males *N* = 250	*p*	Elementary *N* = 615	Secondary *N* = 330	*p*
Bortner scale	Type A	520 (54.6)	391 (55.8)	128 (51.2)	0.240	343 (55.8)	172 (52.1)	0.314
	Type B	432 (45.4)	310 (44.2)	122 (48.8)		272 (44.2)	158 (47.9)	
Gender	Males	250 (26.3)	/	/		142 (23.1)	106 (32.2)	0.003 **
	Females	701 (73.6)	/	/		473 (76.9)	223 (67.8)	
Workplace	Elementary school	615 (65.1)	473 (68)	142 (57.3)	0.003 **	/	/	
	Secondary school	330 (34.9)	223 (32)	106 (42.7)		/	/	
Age (years)	<35	286 (30.4)	210 (30)	76 (30.4)	0.000 ***	191 (31.1)	90 (27.3)	0.000 ***
	36–45	327 (30)	252 (35.9)	75 (30)		220 (35.8)	107 (32.4)	
	46–55	186 (15.6)	147 (21)	39 (15.6)		128 (20.8)	57 (17.3)	
	>56	153 (24)	92 (13.1)	60 (24)		76 (12.4)	76 (23)	
Marital status	Single	207 (21.7)	139 (19.8)	68 (27.2)	0.005 **	128 (20.8)	77 (23.3)	0.009 **
	Married	667 (70.1)	496 (70.8)	171 (68.4)		448 (72.8)	215 (65.2)	
	Divorced/Widowed	78 (8.2)	66 (9.4)	11 (4.4)		39 (6.3)	38 (11.5)	
Children	No	294 (30.9)	199 (28.4)	95 (38)	0.006 **	179 (29.1)	113 (34.2)	0.120
	Yes	658 (69.1)	502 (71.6)	155 (62)		436 (70.9)	217 (65.8)	
Contract	Temporary	76 (8.0)	61 (8.7)	15 (6)	0.229	53 (8.6)	23 (7)	0.442
	Permanent	875 (91.9)	640 (91.3)	234 (94)		561 (91.4)	307 (93)	
Length of work (years)	<10	376 (39.5)	271 (38.7)	105 (42)	0.076	243 (39.5)	128 (38.8)	0.041 *
	11–20	305 (32.0)	239 (34.1)	66 (26.4)		212 (34.5)	93 (28.2)	
	21 and higher	271 (28.5)	191 (27.2)	79 (31.6)		160 (26)	109 (33)	
Overtime work (per week)	Never	912 (95.8)	542 (77.3)	180 (72)	0.203	490 (79.7)	228 (69.1)	0.001 **
	to 10 h	31 (3.3)	152 (21.7)	68 (27.2)		120 (19.5)	99 (30.0)	
	>10 h	9(0.9)	7 (1)	2 (0.8)		5 (0.8)	3 (0.9)	

* *p* < 0.05; ** *p* < 0.01; *** *p* < 0.001.

**Table 2:** Occupational and work–life characteristics in the total study sample stratified by gender, workplace, and behavior type

Occupational and Work/Life Characteristic’s *N* (%).			Gender *N* = 951			Workplace (School Type) *N* = 945			Behavior Type *N* = 952		
		All *N* = 952	Females *N* = 701	Males *N* = 250	*p*	Elementary *N* = 615	Secondary *N* = 330	*p*	Type A *N* = 520	Type B *N* = 432	*p*
Satisfaction with equipment	Very unsatisfied	33 (3.5)	26 (3.7)	7 (2.8)	0.124	22 (3.6)	11 (3.3)	0.895	16 (3.1)	17 (3.9)	0.189
	Unsatisfied	255 (26.8)	181 (25.8)	74 (29,6)		172 (28)	83 (25.2)		150 (28.8)	105 (24.3)	
	Neutral	212 (22.3)	169 (24.1)	43 (17.2)		133 (21.6)	77 (23.3)		118 (22.7)	94 (21.8)	
	Satisfied	425 (44.6)	308 (43.9)	116 (46.4)		270 (43.9)	150 (45.5)		226 (43.5)	199 (46.1)	
	Very satisfied	27 (2.8)	17 (2.4)	10 (4)		18 (2.9)	9 (2.7)		10 (1.1)	17 (1.8)	
Satisfaction with monthly income	Very unsatisfied	76 (8)	54 (7.7)	22 (8.8)	0.202	49 (8)	27 (8.2)	0.825	40 (7.7)	36 (8.3)	0.026 *
	Unsatisfied	335 (35.2)	254 (36.2)	80 (32)		211 (34.3)	123 (37.3)		201(38.7)	134 (31)	
	Neutral	191 (20.1)	138 (19.7)	53 (21.2)		121 (19.7)	67 (20.3)		110 (21.2)	81 (18.8)	
	Satisfied	339 (35.6)	250 (35.7)	89 (35.6)		227 (36.9)	109 (33)		165(31.7)	174 (40.3)	
	Very satisfied	11 (1.2)	5 (0.7)	6 (2.4)		7 (1.1)	4 (1.2)		4 (0.8)	7 (1.6)	
Satisfaction with support of friends/family	Very unsatisfied	24 (2.5)	13 (1.9)	11 (4.4)	0.027 *	16 (2.6)	8 (2.4)	0.032 *	10 (1.9)	14 (3.2)	0.309
	Unsatisfied	24 (2.5)	19 (2.7)	5 (2)		12 (2)	12 (3.6)		14 (2.7)	10 (2.3)	
	Neutral	85 (8.9)	54 (7.7)	31 (12.4)		56 (9.1)	29 (8.8)		43 (8.3)	42 (9.7)	
	Satisfied	476 (50)	361 (51.5)	114 (45.6)		289 (47)	182 (55.2)		274 (52.7)	202 (46.8)	
	Very satisfied	343 (36)	254 (36.2)	89 (35.6)		242 (39.3)	99 (30)		179 (34.4)	164 (38)	
Work-life conflict	Yes	541 (56.8)	401 (57.2)	139 (55.6)	0.715	336 (54.6)	203 (61.5)	0.049 *	310 (59.6)	231 (53.5)	0.066
	No	411 (43.2)	300 (42.8)	111 (44.4)		279 (45.4)	127 (38.5)		210 (40.4)	201 (46.5)	

* *p* < 0.05.

**Table 3:** Job content questionnaire results stratified by gender, workplace, and behavior type

Job Content *N* (%)			Gender *N* = 951			Workplace *N* = 945			Behavior Type *N* = 952		
		All *N* = 952	Females *N* = 701	Males *N* = 250	*p*	Elementary *N* = 615	Secondary *N* = 330	*p*	Type A *N* = 520	Type B *N* = 432	*p*
Working environment Mean (SD)	Job demand	28.36 (4.71)	28.48 (4.64)	27.98 (4.90)	0.150	28.43 (4.80)	28.21 (4.59)	0.489	28.87 (4.83)	27.74 (4.91)	0.000 ***
	Job control	69.73 (7.59)	70.05 (7.55)	68.83 (7.66)	0.029 *	69.64 (7.94)	69.8 (6.94)	0.700	70.14 (7.75)	69.24 (7.35)	0.071
	Decision latitude	32.26 (4.29)	32.25 (4.26)	32.27 (4.36)	0.947	32.17 (4.50)	32.44 (3.88)	0.362	32.51 (4.26)	31.96 (4.30)	0.051
	Skill-discretion	37.47 (4.92)	37.80 (4.86)	36.56 (5.00)	0.001 **	37.47 (5.09)	37.41 (4.61)	0.842	37.63 (6.06)	37.28 (4.75)	0.277
	Supervisor support	11.80 (2.39)	11.86 (2.34)	11.62 (2.53)	0.184	11.89 (2.29)	11.62 (2.56)	0.098	11.67 (2.40)	11.95 (2.37)	0.069
	Co-workers support	11.55 (1.82)	11.62 (1.79)	11.36 (1.88)	0.054	11.63 (1.84)	11.39 (1.76)	0.054	11.46 (1.94)	11.67 (1.66)	0.062
Job strain N (%)	Indicated	132 (13.9)	94 (13.4)	38 (15.2)	0.551	90 (14.6)	42 (12.7)	0.479	88 (16.9)	44 (10.2)	0.004 **
	Not indicated	820 (86.1)	607 (86.6)	212 (84.8)		525 (85.4)	288 (87.3)		432 (83.2)	338 (89.8)	

* p < 0.05; ** *p* < 0.01; *** *p* < 0.001.

**Table 4:** Scores on burnout syndrome scales stratified by individual and socio-demographic characteristics

Worker Characteristics *N* (%)	Emotional Exhaustion-EE				Depersonalization-DP				Personal Accomplishment-PA			
	Low	Moderate	High	*p*	Low	Moderate	High	*p*	Low	Moderate	High	*p*
All teachers	747 (78.5)	156 (16.4)	49 (5.1)		833 (87.5)	83 (8.7)	36 (3.8)		212 (22.3)	245 (25.7)	495 (52)	
Type behavior												
Type A	391 (52.3)	100 (64.1)	29 (59.2)	0.022 *	445 (53.4)	49 (59)	26 (72.2)	0.060	122 (57.5)	145 (59.2)	253 (51.1)	0.072
Type B	356 (47.7)	56 (35.9)	20 (40.8)		388 (46.6)	34 (41)	10 (27.8)		90 (42.5)	100 (40.8)	242 (48.9)	
Gender												
Males	184 (24.6)	47 (30.3)	19 (38.8)	0.043 *	219 (26.3)	17 (20.5)	14 (40)	0.089	47 (22.2)	73 (29.8)	130 (26.3)	0.182
Females	563 (75.4)	108 (69.7)	30 (61.2)		614 (73.7)	66 (79.5)	21 (60)		165 (77.8)	172 (70.2)	364 (73.7)	
Age group (years)												
<35	237 (31.7)	36 (23.1)	13 (26.5)	0.012 *	258 (31)	20 (24.1)	8 (22.2)	0.165	49 (23.1)	64 (26.1)	173 (34.9)	0.000 ***
36–45	258 (34.5)	56 (35.9)	13 (26.5)		288 (34.6)	23 (27.7)	16 (44.4)		67 (31.6)	83 (33.9)	177 (35.8)	
46–55	149 (19.9)	27 (17.3)	10 (20.5)		160 (19.2)	21 (25.3)	5 (13.9)		49 (23.1)	63 (25.7)	74 (14.9)	
>56	103 (13.8)	37 (23.7)	13 (26.5)		127 (15.2)	19 (22.9)	7 (19.4)		47 (22.2)	35 (14.3)	71 (14.3)	
Marital status												
Single	159 (21.3)	35 (22.4)	13 (26.5)	0.068	189 (22.7)	10 (12)	8 (22.2)	0.000 ***	42 (19.8)	48 (19.6)	117 (23.6)	0.053
Married	535 (71.6)	104 (66.7)	28 (57.1)		586 (70.3)	56 (67.5)	25 (69.4)		143 (67.5)	178 (72.6)	346 (69.9)	
Divorced/widowed	53 (7.1)	17 (10.9)	8 (16.3)		58 (7)	17 (20.5)	3 (8.3)		27 (12.7)	19 (7.8)	32 (6.5)	
Children												
No	226 (30.3)	51 (32.7)	17 (34.7)	0.701	266 (31.9)	18 (21.7)	10 (27.8)	0.144	57 (26.9)	71 (29)	166 (33.5)	0.163
Yes	521 (69.7)	105 (67.3)	32 (65.3)		567 (68.1)	65 (78.3)	26 (72.2)		155 (73.1)	174 (71)	329 (66.5)	
School type												
Elementary	491 (66.4)	91 (58.3)	33 (67.3)	0.152	538 (65.1)	47 (56.6)	30 (83.3)	0.019 *	124 (58.5)	167 (68.4)	324 (66.3)	0.062
Secondary	249 (33.6)	65 (41.7)	16 (32.7)		288 (34.9)	36 (43.4)	6 (16.7)		88 (41.5)	77 (31.6)	165 (33.7)	
Contract type												
Temporary	59 (7.9)	15 (9.7)	2 (4.1)	0.443	72 (8.7)	3 (3.6)	1 (2.8)	0.136	13 (6.1)	11 (4.5)	52 (10.5)	0.009 **
Permanent	688 (92.1)	140 (90.3)	47 (95.9)		760 (91.3)	80 (96.4)	35 (97.2)		199 (93.9)	234 (95.5)	442 (89.5)	
Length of work (years)												
<10	306 (41)	54 (34.6)	16 (32.7)	0.068	337 (40.5)	26 (31.3)	13 (36.1)	0.006 **	60 (28.3)	85 (34.7)	231 (46.7)	0.000 ***
11–20	245 (32.8)	46 (29.5)	14 (28.6)		276 (33.1)	20 (24.1)	9 (25)		64 (30.2)	89 (36.3)	152 (30.7)	
>20	196 (26.2)	56 (35.9)	19 (38.7)		220 (26.4)	37 (44.6)	14 (38.9)		88 (41.5)	71 (29)	112 (22.6)	
Overtime work (per week)												
Never	608 (81.4)	82 (52.6)	32 (65.3)	0.000 ***	657 (78.9)	42 (50.6)	23 (63.9)	0.000 ***	145 (68.4)	182 (74.3)	395 (79.8)	0.002 **
to 10 h	133 (17.8)	74 (47.4)	14 (28.6)		167 (20)	41 (49.4)	13 (36.1)		67 (31.6)	61 (24.9)	93 (18.8)	
>10 h	6 (0.8)	0 (0)	3 (6.1)		9 (1.1)	0 (0)	0 (0)		0 (0)	2 (0.8)	7 (1.4)	

* *p* < 0.05; ** *p* < 0.01; *** *p* < 0.001.

**Table 5:** Burnout scores stratified by work/life and occupational characteristics

Work/Life and Occupational Characteristics		Emotional Exhaustion-EE		Depersonalization-DP		Personal Accomplishment-PA	
		Med (Min-Max)	*p*	Med (Min-Max)	*p*	Med (Min-Max)	*p*
Satisfaction with equipment	Very unsatisfied	6.00 (0.00–50)	0.000 ***	0.00 (0.00–17)	0.000 ***	41.00 (0.00–48)	0.000 ***
	Unsatisfied	10.00 (0.00–42)		2.00 (0.00–25)		38.00 (4–48)	
	Neutral	9.50 (0.00–40)		1.00 (0.00–19)		37.00 (11–48)	
	Satisfied	6.00 (0.00–42)		0.00 (0.00–25)		40.00 (3–48)	
	Very satisfied	4.00 (0.00–33)		0.00 (0.00–18)		43.00 (12–48)	
Satisfaction with monthly income	Very unsatisfied	10.00 (0.00–20)	0.000 ***	2.00 (0.00–17)	0.000 ***	33.50 (0.00–48)	0.000 ***
	Unsatisfied	9.00 (0.00–42)		1,00 (0.00–25)		38.00 (3–48)	
	Neutral	11.00 (0.00-38)		2.00 (0.00-20)		37.00 (11–48)	
	Satisfied	5.00 (0.00–34)		0.00 (0.00–20)		42.00 (4–48)	
	Very satisfied	7.00 (0.00–28)		0.00 (0.00–16)		44.00 (28–48)	
Satisfaction with support of friends/family	Very unsatisfied	14.00 (0.00–26)	0.000 ***	0.00 (0.00–16)	0.000 ***	41.00 (0.00–48)	0.000 ***
	Unsatisfied	19.00 (6–30)		7.00 (0.00–15)		25.00 (15–40)	
	Neutral	18.00 (0.00–50)		5.00 (0.00–21)		34.00 (3–48)	
	Satisfied	8.00 (0.00–42)		1.00 (0.00–25)		38.00 (4–48)	
	Very satisfied	6.00 (0.00–40)		0.00 (0.00–17)		41.00 (12–48)	
Work-life conflict (last 3 weeks)	Yes	11.00 (0.00–50)	0.000 ***	2.00 (0.00–25)	0.000 ***	36.00 (10–48)	0.000 ***
	No	5.00 (0.00–35)		0.00 (0.00–21)		41.00 (0.00–48)	
Job strain	“Indicated“	16.50 (0.00–42)	0.000 ***	4.00 (0.00–20)	0.000 ***	33.00 (0.00–48)	0.000 ***
	“Not indicated”	7.00 (0.00–50)		1.00 (0.00–25)		40.00 (3–48)	

*** *p* < 0.001.

**Table 6:** Factors associated with the MBI-HSS score (hierarchical linear regression)

Independent Variables	Emotional Exhaustion STAND β				Depersonalization Stand β				Personal Accomplishment Stand β			
	Step I	Step II	Step III	Step IV	Step I	Step II	Step III	Step IV	Step I	Step II	Step III	Step IV
Type behavior	−0.119 ***	−0.131 ***	−0.098 **	−0.074 **	−0.104 **	−0.108 **	−0.087 **	−0.067 *	0.081 *	0.086 **	0.061 *	0.053
Gender		−0.060	−0.055	−0.045		−0.060	−0.050	−0.035		0.003	−0.006	−0.031
Age		0.035	0.064	0.053		−0.054	−0.041	−0.050		0.036	0.028	0.023
Marital status		0.102 *	0.082 *	0.065		0.199 *	0.074	0.047		−0.054	−0.028 *	−0.005
Children (dichotomy)		−0.114 **	−0.111 **	−0.083 *		−0.053	−0.045	−0.016		0.080	0.075	0.045
Workplace		0.044	0.021	0.026		−0.009	−0.030	−0.019		−0.067 *	−0.042	−0.053
Contract		0.006	0.013	−0.010		0.016	0.027	0.008		−0.018	−0.030	−0.015
Length of work		0.107 *	0.003	−0.005		0.139 *	0.049	0.038		−0.195 ***	−0.103 *	−0.073
Overtime work (dichotomy)		0.063 *	0.042	0.050		0.030	0.027	0.015		−0.016	0.031	0.023
Work-life conflict			0.276 ***	0.210 ***			0.172 ***	0.113 ***			−0.210 ***	−0.136 ***
Satisfaction with equipment			−0.147 ***	−0.127 ***			−0.033	−0.020			0.005	−0.015
Satisfaction with salary			−0.065 *	−0.035			−0.080 *	−0.063			0.109 **	0.089 **
Satisfaction with support of friends/family			−0.145 ***	−0.088 ***			−0.209 ***	−0.163 ***			0.203 ***	0.123 ***
Skill discretion				−0.136 ***				−0.214 ***				0.263 ***
Decision-making authority				0.090 **				−0.156 ***				0.004
Job demand				0.186 ***				0.187 ***				−0.142 ***
Supervisor support				−0.089 **				−0.065				0.059
Co-workers’ support				−0.042				−0.051				0.044
R2	0.014 ***	0.052 ***	0.209 ***	0.281 ***	0.011 **	0.034 *	0.136 ***	0.212 ***	0.007 *	0.043 ***	0.163 ***	0.269 ***
ΔR2		0.038	0.157	0.072		0.023	0.102	0.076		0.036	0.120	0.106

* *p* < 0.05; ** *p* < 0.01, *** *p* < 0.001.

## References

[1] Maslach C., Schaufeli W.B., Leiter M. (2001). Job Burnout. Annu. Rev. Psychol..

[2] García-Arroyo J.A., Segovia A.O., Peiró J. (2019). Meta-analytical review of teacher burnout across 36 societies: The role of national learning assessments and gender egalitarianism. Psychol. Health.

[3] Salvagioni D.A.J., Melanda F.N., Mesas A.E., González A.D., Gabani F.L., De Andrade S.M. (2017). Physical, psychological and occupational consequences of job burnout: A systematic review of prospective studies. PLoS ONE.

[4] Schaufeli W.B., Leiter M., Maslach C. (2009). Burnout: 35 years of research and practice. Career Dev. Int..

[5] Lastovkova A., Carder M., Rasmussen H.M., Sjoberg L., De Groene G.J., Sauni R., Vevoda J., Vevodova S., Lasfargues G., Svartengren M. (2018). Burnout syndrome as an occupational disease in the European Union: An exploratory study. Ind. Health.

[6] Goetz K., Loew T., Hornung R., Cojocaru L., Lahmann C., Tritt K. (2013). Primary Prevention Programme for Burnout-Endangered Teachers: Follow-Up Effectiveness of a Combined Group and Individual Intervention of AFA Breathing Therapy. Evidence-Based Complement. Altern. Med..

[7] Alarcon G.M., Eschleman K.J., Bowling N.A. (2009). Relationships between personality variables and burnout: A meta-analysis. Work. Stress.

[8] Antoniou A.-S., Polychroni F., Vlachakis A.-N. (2006). Gender and age differences in occupational stress and professional burnout between primary and high-school teachers in Greece. J. Manag. Psychol..

[9] Antoniou A.-S., Ploumpi A., Ntalla M. (2013). Occupational Stress and Professional Burnout in Teachers of Primary and Secondary Education: The Role of Coping Strategies. Psychology.

[10] Arvidsson I., Håkansson C., Karlson B., Björk J., Persson R. (2016). Burnout among Swedish school teachers-a cross-sectional analysis. BMC Public Health.

[11] Martocchio J.J., O’Leary A.M. (1989). Sex Differences in Occupational Stress: A Meta-Analytic Review. J. Appl. Psychol..

[12] Purvanova R.K., Muros J.P. (2010). Gender differences in burnout: A meta-analysis. J. Vocat. Behav..

[13] Schwarzer R., Schmitz G.S., Tang C. (2000). Teacher Burnout in Hong Kong and Germany: A Cross-Cultural Validation of the Maslach Burnout Inventory. Anxiety Stress. Coping.

[14] Burke R.J., Greenglass E. (1993). Work Stress, Role Conflict, Social Support, and Psychological Burnout among Teachers. Psychol. Rep..

[15] Fiorilli C., Gabola P., Pepe A., Meylan N., Curchod-Ruedi D., Albanese O., Doudin P.-A. (2015). The effect of teachers’ emotional intensity and social support on burnout syndrome. A comparison between Italy and Switzerland. Eur. Rev. Appl. Psychol..

[16] Harding S., Morris R., Gunnell D., Ford T., Hollingworth W., Tilling K., Evans R., Bell S.L., Grey J., Brockman R. (2019). Is teachers’ mental health and wellbeing associated with students’ mental health and wellbeing?. J. Affect. Disord..

[17] Herman K.C., Hickmon-Rosa J., Reinke W. (2017). Empirically Derived Profiles of Teacher Stress, Burnout, Self-Efficacy, and Coping and Associated Student Outcomes. J. Posit. Behav. Interv..

[18] Kreso A.P. (2008). The War and Post-War Impact on the Educational System of Bosnia and Herzegovina. Int. Rev. Educ..

[19] Paleksić V., Ubović R., Popović M. (2015). Personal Characteristics and Burnout Syndrome among Teachers of Primary and Secondary Schools. Scr. Medica.

[20] Roberts D.L., Shanafelt T.D., Dyrbye L.N., West C.P. (2014). A national comparison of burnout and work-life balance among internal medicine hospitalists and outpatient general internists. J. Hosp. Med..

[21] Jovanović D., JakovljeviĆ B., Paunovic K., Grubor D. (2006). Importance of personality traits and psychosocial factors for the development of coronary heart disease. Vojn. Pregl..

[22] Edwards J., Baglioni A.J., Cooper C. (1990). The psychometric properties of the Bortner Type A Scale. Br. J. Psychol..

[23] Jepson E., Forrest S. (2006). Individual contributory factors in teacher stress: The role of achievement striving and occupational commitment. Br. J. Educ. Psychol..

[24] Bernotaite L., Malinauskiene V. (2017). Workplace bullying and mental health among teachers in relation to psychosocial job characteristics and burnout. Int. J. Occup. Med. Environ. Health.

[25] Blom V., Bodin L., Bergström G., Hallsten L., Svedberg P. (2013). The Importance of Genetic and Shared Environmental Factors for the Associations between Job Demands, Control, Support and Burnout. PLoS ONE.

[26] Borrelli I., Benevene P., Fiorilli C., D’Amelio F., Pozzi G. (2014). Working conditions and mental health in teachers: A preliminary study. Occup. Med..

[27] Wang Y., Ramos A., Wu H., Liu L., Yang X., Wang J., Wang L. (2014). Relationship between occupational stress and burnout among Chinese teachers: A cross-sectional survey in Liaoning, China. Int. Arch. Occup. Environ. Health.

[28] Sale J.E.M., Kerr M.S. (2002). The psychometric properties of Karasek’s demand and control scales within a single sector: Data from a large teaching hospital. Int. Arch. Occup. Environ. Health.

[29] Matejic B., Milenović M., Tepavcevic D.K., Simic D., Pekmezović T., Worley J.A. (2015). Psychometric Properties of the Serbian Version of the Maslach Burnout Inventory-Human Services Survey: A Validation Study among Anesthesiologists from Belgrade Teaching Hospitals. Sci. World J..

[30] Borg M.G., Riding R.J. (1991). Occupational Stress and Satisfaction in Teaching. Br. Educ. Res. J..

[31] Bauer J., Stamm A., Virnich K., Wissing K., Müller U., Wirsching M., Schaarschmidt U. (2005). Correlation between burnout syndrome and psychological and psychosomatic symptoms among teachers. Int. Arch. Occup. Environ. Health.

[32] Greenglass E.R., Burke R.J., Konarski R. (1997). The impact of social support on the development of burnout in teachers: Examination of a model. Work. Stress.

[33] Kokkinos C.M. (2007). Job stressors, personality and burnout in primary school teachers. Br. J. Educ. Psychol..

[34] Caplan R.D., Jones K.W. (1975). Effects of Work Load, Role Ambiguity, and Type A Personality on Anxiety, Depression, and Heart Rate. J. Appl. Psychol..

[35] Spector P.E., O’Connell B.J. (1994). The contribution of personality traits, negative affectivity, locus of control and Type A to the subsequent reports of job stressors and job strains. J. Occup. Organ. Psychol..

[36] Greenglass E.R., Burke R.J. (1988). Work and Family Precursors of Burnout in Teachers: Sex Differences I. Sex Roles..

[37] González-Morales M.G., Molina I.R., Peiró J. (2010). A longitudinal study of coping and gender in a female-dominated occupation: Predicting teachers’ burnout. J. Occup. Health Psychol..

[38] Kamtsios S. (2018). Burnout syndrome and stressors in different stages of teachers professional development. Hell. J. Psychol..

[39] Russell D.W., Altmaier E., Van Velzen D. (1987). Job-Related Stress, Social Support, and Burnout Among Classroom Teachers. J. Appl. Psychol..

[40] Assessing Your Behavior Pattern. https://papers.ssrn.com/sol3/papers.cfm?abstract_id=1280666.

[41] Mojsa-Kaja J., Golonka K., Marek T. (2015). Job burnout and engagement among teachers–Worklife areas and personality traits as predictors of relationships with work. Int. J. Occup. Med. Environ. Health.

